# Novel omnipolar mapping technology for effective superior vena cava isolation: A randomized clinical trial

**DOI:** 10.1002/joa3.70007

**Published:** 2025-01-23

**Authors:** Naoto Oguri, Yousaku Okubo, Naoki Ishibashi, Junji Maeda, Takumi Sakai, Yukimi Uotani, Motoki Furutani, Shogo Miyamoto, Shunsuke Miyauchi, Sho Okamura, Takehito Tokuyama, Noboru Oda, Yukiko Nakano

**Affiliations:** ^1^ Department of Cardiovascular Medicine, Graduate School of Biomedical and Health Sciences Hiroshima University Hiroshima Japan

**Keywords:** atrial fibrillation, catheter ablation, high density mapping, omnipolar mapping technology, superior vena cava isolation

## Abstract

**Background:**

Successful isolation of the superior vena cava (SVC) using a functional conduction block between the right atrium (RA) and SVC has been documented. However, a comparison of this approach with the conventional method (CM) of circumferential ablation of the RA‐SVC junction, based on angiography, remains unexplored.

**Objective:**

In this study, we employed the innovative omnipolar mapping technology (OT) to discern the RA‐SVC connection and compared clinical outcomes with those from CM.

**Methods:**

Sixty‐two patients undergoing SVC isolation were randomly assigned in a 1:1 ratio to either the OT or CM group. No significant differences in the baseline characteristics were observed between the two groups. We assessed the efficacy and safety of both groups.

**Results:**

Both groups showed comparable acute success rates (96%) in SVC isolation, but the procedure in the OT group required fewer radiofrequency (RF) applications (13.6 ± 6.0 vs. 19.8 ± 10.9, *p* = .046) and shorter procedure time (9.6 ± 6.8 min vs. 14.3 ± 6.8 min, *p* = .007). The overall absorbed dose was notably lower in the OT group (69.6 ± 47.6 mGy vs. 90.3 ± 30.3 mGy, *p* = .023).

**Conclusions:**

The OT enhances the efficacy of SVC isolation, requiring fewer RF applications and reducing procedure time compared to conventional treatment methods.

## Graphical abstract

SVC isolation is sometimes difficult because of avoidance of phrenic nerve and sinus node injury. Omnipolar mapping technology (OT) could enhance the efficacy of SVC isolation, requiring fewer RF applications and reducing procedure duration.

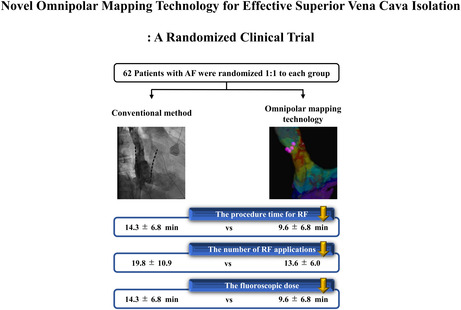

## 1 INTRODUCTION

Pulmonary vein isolation (PVI) is a fundamental component of catheter ablation for atrial fibrillation (AF), given that spontaneous ectopy originating from the pulmonary veins (PVs) is recognized as a significant instigator of paroxysmal AF.^1^ Nonetheless, AF can also be triggered by non‐pulmonary vein (non‐PV) ectopy, which can be found in areas such as the left atrial (LA) posterior wall, crista terminalis, eustachian ridge region, coronary sinus ostium, ligament of Marshall, and superior vena cava (SVC). Specifically, the SVC has been recognized as a significant non‐PV source of AF, identifiable in roughly 5% of patients undergoing AF ablation.^2^ Prior research indicates that SVC isolation may enhance the long‐term success of catheter ablation.^3^ However, the procedure often presents challenges because of the necessity to circumvent potential injury to the phrenic nerve (PN) and sinus node (SN) injury.^4^, ^5^, ^6^

Recent studies have reported the efficacy of SVC isolation through the use of a functional conduction block between the right atrium (RA) and SVC.^7^, ^8^, ^9^ These studies have shown a reduction in the number of radiofrequency (RF) applications needed for SVC isolation in instances where conduction block lines are present, as compared to those without such lines. Despite the anticipation that this innovative approach to SVC isolation will offer safer and more effective isolation, there have been no comparative studies between this technique and the conventional method (CM), which performs circumferential ablation at the RA‐SVC junction, guided only by angiography. Furthermore, prior research has established a three‐dimensional electroanatomical mapping of SVC and SN using bipolar electrograms, and there has been no report using Omnipolar mapping technology (OT) for SVC isolation. OT represents an innovative tool designed to capture electrogram signals, irrespective of catheter orientation, by amalgamating signals from three electrodes to form triangular cliques.^10^

In the current study, we used OT to identify the RA‐SVC connection and the SN location, comparing these clinical outcomes with those of CM.

## 2 METHODS

### 2.1 Study design and patient population

This prospective, randomized clinical trial enrolled 330 patients who underwent radiofrequency catheter ablation (RFCA) for AF at Hiroshima University Hospital from January 2022 to January 2023. Prior to the procedure, patients who had previously undergone SVC isolation (*n* = 11), those with an implanted pacemaker (*n* = 3), and those with bradycardia arrhythmias (*n* = 6) were excluded. We also excluded patients without non‐PV foci from the SVC at initial AF ablation during the procedure (*n* = 248). In total, 62 patients were recruited and randomly allocated in a 1:1 ratio to either the OT group or the CM group.

The study received approval from the Institutional Ethics Committee of Hiroshima University's Graduate School of Biomedical Science (approval no. C2021‐0339, registered on September 30, 2020). It was conducted in strict adherence to the principles outlined in the Declaration of Helsinki. All participants furnished their written informed consent.

### 2.2 SVC mapping and ablation procedure

Prior to the procedure, antiarrhythmic medications were discontinued for a duration equivalent to at least five half‐lives of the drug. All patients underwent a minimum of 1 month of efficacious anticoagulation therapy, coupled with transesophageal echocardiography, to rule out the presence of atrial thrombosis prior to the procedure. Following the transseptal access, an activation clotting time exceeding 250 s was consistently sustained throughout the procedure. In every patient, PVI was executed prior to the mapping of the SVC and RA. Successful PVI was verified through the demonstration of entrance and exit blocks using a circular catheter.

In the OT group, electrophysiological mapping of the SVC and RA during sinus rhythm was created using a high‐density mapping catheter (Advisor™ HD‐Grid Mapping Catheter, Abbott, St. Paul, MN) and an EnSite™ X EP system (Abbott, St. Paul, MN) to identify the SN location and the RA‐SVC junction. Electrograms exhibiting a peak‐to‐peak voltage below <0.05 mV were categorized as either scar tissue or electrically inactive. The creation of bipolar voltage mapping was facilitated by the superior duplicate algorithm, which selectively identifies the local bipolar electrogram with the highest voltage from two proximate pairs. Conversely, the electrophysiological characteristics of omnipolar mappings, including voltage and directionality, were ascertained based on signals derived from a triad of contiguous electrodes. We characterized the SN as the site of earliest activation, as determined by electrophysiological mapping of the RA and SVC. The length of the myocardial sleeve in the SVC was determined by measuring the distance from the uppermost point of the SN to the highest observable level of the SVC potentials. We have configured the color contour to display 15 distinct colors, each representing a 10 ms interval, ranging from the earliest to the latest activation site. The functional conduction block between the SVC and RA was defined as the line where the spectrum spanned ≥ three colors within a short distance.

In the CM group, RA‐SVC angiography was conducted via manual injection to accurately pinpoint the RA‐SVC junction. Based on prior studies, we identified the intersection of the convex RA wall and the straight SVC wall on the angiogram as the radiological RA‐SVC junction, as referenced in the citation.^11^

Prior to SVC isolation, we initially conducted high‐output pacing (5.0 V/0.5 ms) using an ablation catheter to ascertain the precise location where the PN was captured. Subsequently, we positioned a circular mapping catheter superior to the RA‐SVC junction. This allowed us to observe SVC potentials in both study groups.

**FIGURE 1:**
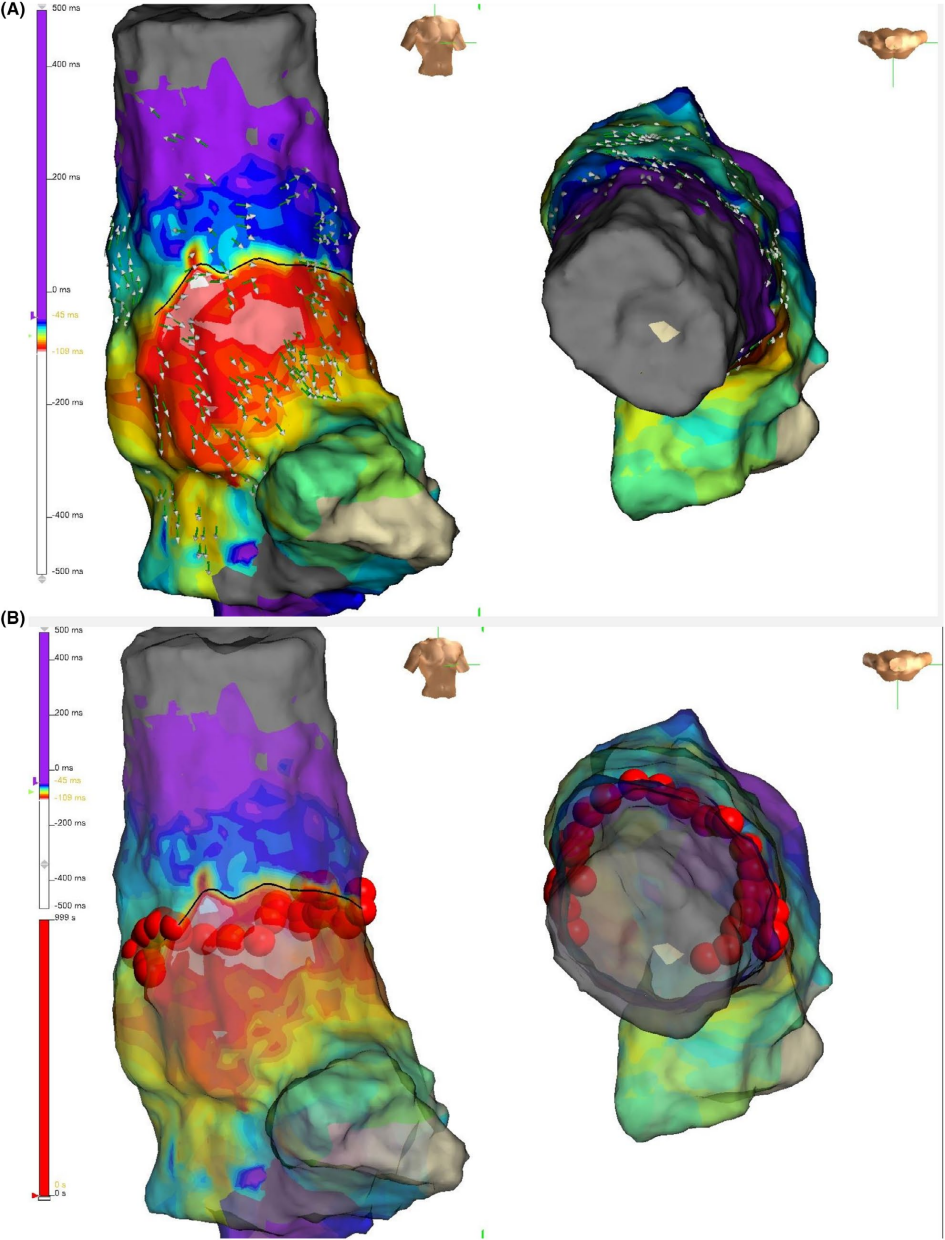
An illustration of the OT‐guided SVC isolation. (A) The left‐hand map presents an anteroposterior view (AP), while the right‐hand map provides an overhead perspective. The direct conduction block line (Black line) was observed running obliquely on the anterolateral aspect, positioned just superior to the SN. (B) RF applications (Red tags) were delivered to connect the open end of the conduction block line. OT, omnipolar mapping technology; RF, radiofrequency; SN, sinus node; SVC, superior vena cava.

In the OT group, RF applications were administered along the trajectory linking the open extremities of the conduction block line. (Figure 1) Conversely, in the CM group, RF applications were administered in a circular pattern, approximately 5–10 mm above the radiographic junction of the RA‐SVC. Isolation of the SVC was executed using a point‐by‐point technique. This procedure employed an 8‐F, 4‐mm flexible irrigated‐tip catheter with an interelectrode spacing of 2 to 2‐2 mm (TactiCath™ SE Ablation Catheter, Abbott, St. Paul, MN). RF energy was administered at each location with a power of 30 W, a peak temperature of 42°C, and a targeted Lesion Size Index (LSI) value of 4. The irrigation was administered at a flow rate of 17 mL/min when the temperature was below 42°C and at a rate of 30 mL/min when the temperature exceeded 42°C. The successful isolation of the SVC was validated through the confirmation of a bidirectional conduction block between the RA and the SVC. In this study, four operators with substantial experience in SVC isolation performed all ablation procedures.

### 2.3 Study endpoint

The primary objective of this study was to evaluate the success rate of SVC isolation. In the CM group, the procedure was deemed unsuccessful if SVC isolation could not be achieved through conventional techniques, regardless of whether subsequent application of OT technology led to successful SVC isolation. The secondary endpoint encompassed parameters such as the duration of the procedure, fluoroscopy time and dosage, the count of RF applications, and the cumulative RF energy expended. The procedural duration was segmented into three distinct phases as detailed below: (1) The comprehensive duration of the SVC isolation procedure, commencing from the initiation of RA‐SVC mapping to the finalization of SVC isolation. (2) The duration of the mapping procedure, commencing from the initiation of RA‐SVC activation mapping or RA‐SVC angiography and concluding immediately prior to the onset of ablation. (3) The duration of the RF procedure, spanning from the conclusion of mapping to the successful isolation of the SVC. The safety outcomes evaluated encompassed complications such as PN injury, SN injury, SVC stenosis, and cardiac tamponade.

### 2.4 Statistical analysis

At the beginning of the present study, the completion rate of SVC isolation for determining sample size was obtained from a combination of prior trials, and we estimated the rate was 96% in the OT group, while it was 90% in the CM group. The study was sized to provide 80% power to show the non‐inferiority of SVC isolation using OT compared with the conventional method in a test comparing the primary outcome, assuming a non‐inferiority margin of 0.10 and a one‐tailed alpha of 0.05. The planned sample size per group was 32 patients.

Continuous variables were presented as either mean ± SD or median accompanied by the interquartile range, while categorical variables were expressed as proportions. Descriptive statistics were used to describe the baseline characteristics in each arm with *χ* ^2^ tests for binomial and categorical data, unpaired two‐tailed *t*‐tests for normally distributed continuous variables, and Mann–Whitney tests for skewed continuous variables. The primary analysis involved an unadjusted comparison of the primary outcome, conducted using a *χ* ^2^ test. The secondary outcomes were evaluated using unpaired two‐tailed *t*‐tests for continuous variables with normal distribution, and Mann–Whitney tests were employed for continuous variables with skewed distribution. All tests were conducted bilaterally, with results deemed statistically significant at *p* < .05. Statistical analyses were conducted using JMP software, version 15.0, from SAS Institute, Cary, North Carolina.

## 3 RESULTS

### 3.1 Baseline characteristics

**FIGURE 2:**
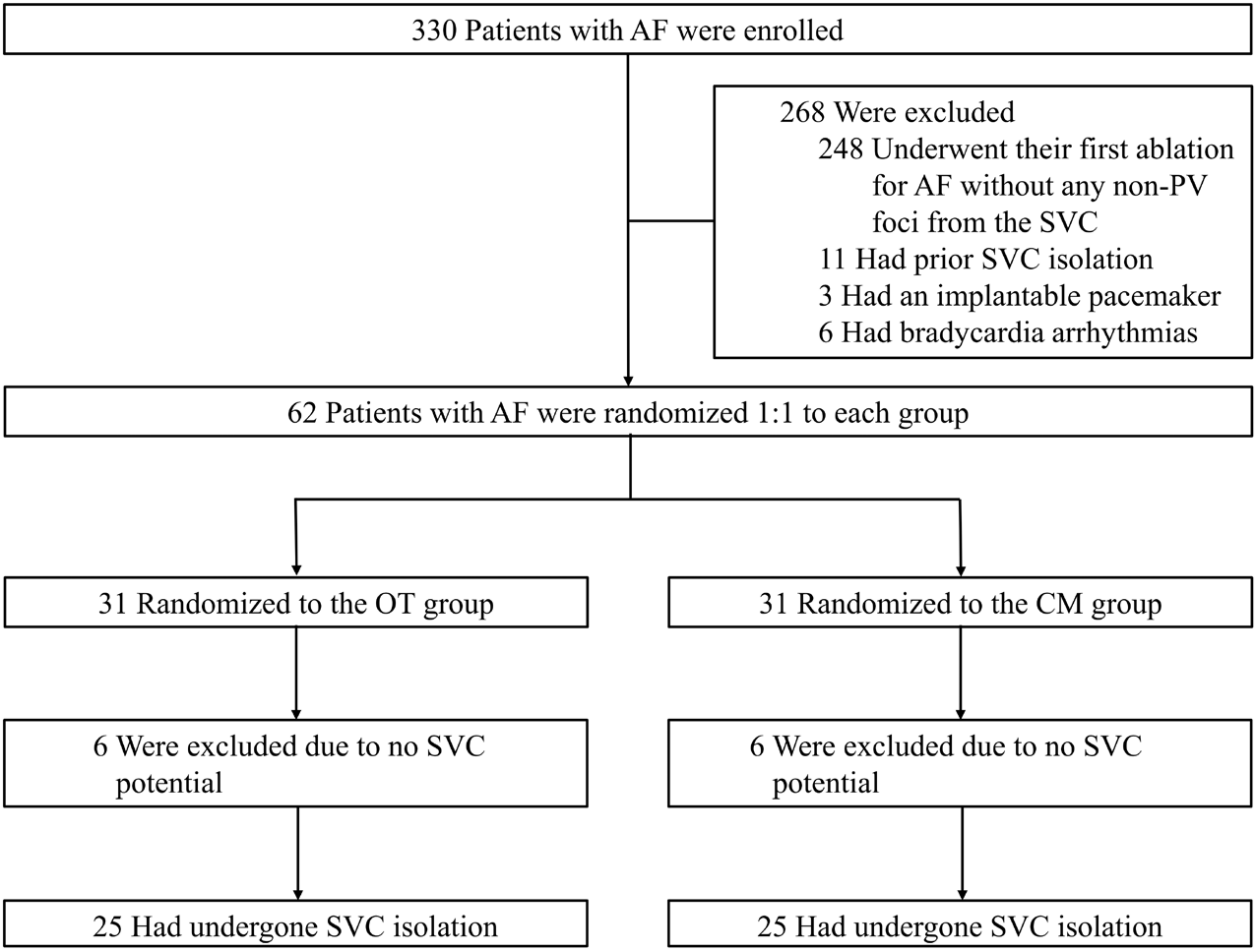
Patient allocation and progression within the current study. AF, atrial fibrillation; CM, conventional method; OT, omnipolar mapping technology; SVC, superior vena cava.

In the current research, a total of 62 participants were randomly allocated in a 1:1 ratio to either the OT group (*N* = 31) or the CM group (*N* = 31). In each group, six participants were excluded because of the brevity or absence of the SVC sleeve. Of the remaining 25 individuals in each group, they were ultimately subjected to analysis. (Figure 2) The baseline characteristics are comprehensively outlined in Table 1. The average age of patients was 68.8 ± 10.2 years in the OT group and 67.2 ± 11.0 years in the CM group, respectively. In the OT group, paroxysmal AF was observed in 68.0% of cases, while in the CM group, it was present in 64.0% of cases. SVC foci were observed in four patients in the OT group, and six patients were in the CM group. The median duration from the onset of AF to participant enrollment in this study was 92.0 months. No significant differences were observed between the two groups.

**TABLE 1:** Baseline characteristics of the study population.

Characteristic	Total (*N* = 50)	OT group (*N* = 25)	CM group (*N* = 25)	*P* value
Age, years	68.0 ± 10.6	68.8 ± 10.2	67.2 ± 11.0	.650
Female, %	36 (72.0)	18 (72.0)	18 (72.0)	1.000
Body mass index^a^	23.9 ± 3.6	24.8 ± 4.4	23.1 ± 2.4	.092
Hypertension, %	29 (58.0)	16 (64)	12 (52.2)	.776
Diabetes mellitus, %	5 (10.0)	4 (16.0)	1 (4.0)	.352
Previous stroke or transit ischemic attack, %	3 (6.0)	2 (8.0)	1 (4.0)	1.000
Congestive heart failure, %	6 (12.0)	3 (12.0)	3 (12.0)	1.000
CHADS2 score	1.2 ± 1.1	1.4 ± 1.0	1.1 ± 1.1	.272
Paroxysmal AF, %^b^	33 (66.0)	17 (68.0)	16 (64.0)	.775
SVC foci, %	10 (20.0)	4 (16.0)	6 (24.0)	.73
Duration from Initial AF diagnosis, month	92.0 ± 98.4	103.0 ± 117.8	80.6 ± 74.9	.743
Left atrial diameter, mm	40.6 ± 5.9	41.4 ± 6.3	39.8 ± 5.5	.320
LVEF, %	59.3 ± 8.1	58.8 ± 10.3	59.8 ± 4.9	.631

*Note*: The values are the means ± SDs, *n* (%), or median [interquartile range] as appropriate. CHADS2 score is a measure of risk of stroke in which congestive heart failure, hypertension, age of 75 years or older, and diabetes mellitus are each assigned 1 point, and previous stroke or transient ischemic attack is assigned 2 points.

Abbreviations: AF, atrial fibrillation; CM, conventional method; LVEF, left ventricular ejection fraction; OT, omnipolar mapping technology; SVC, superior vena cava.

^a^ Calculated as weight in kilograms divided by height in meters squared.

^b^ Paroxysmal AF was defined as AF that terminates spontaneously or with intervention within 7 days of onset.

### 3.2 Primary and secondary endpoints

The clinical outcomes of the current study are succinctly encapsulated in Table 2. In each group, there was one instance where SVC isolation could not be achieved, resulting in a success rate of 96% for both groups. In both cases, residual SVC potentials were observed at the site of PN capture. To prevent potential PN injury, the procedure was discontinued.

**TABLE 2:** Comparison of procedural findings of SVC isolation between the OT group and the CM group.

Variable	OT group (*N* = 25)	CM group (*N* = 25)	*P* value
Successful SVC isolation, %	24 (96.0)	24 (96.0)	1.000
Phrenic nerve injury, %	1 (4.0)	1 (4.0)	1.000
Sinus node injury, %	0 (0.0)	0 (0.0)	1.000
Cardiac tamponade, %	0 (0.0)	0 (0.0)	1.000
SVC stenosis, %	0 (0.0)	0 (0.0)	1.000
Total procedural time for SCV isolation, min	17.2 ± 10.0	20.1 ± 8.4	.132
Procedural time for mapping, min	6.6 ± 2.8	5.3 ± 3.2	.051
Procedural time for RF, min	9.6 ± 6.8	14.3 ± 6.8	.007
Number of RF applications	13.6 ± 6.0	19.8 ± 10.9	.046
Total RF time, min	5.1 ± 2.9	6.8 ± 3.6	.036
Total RF energy, J	7104 ± 4410	10 088 ± 5760	.039
Fluoroscopy time to SVC isolation, min	7.1 ± 4.1	7.6 ± 3.7	.574
Fluoroscopy dose, mGy	69.6 ± 47.6	90.3 ± 30.3	.023

*Note*: The values are the means ± SDs, *n* (%), or median [interquartile range] as appropriate. SVC isolation is sometimes difficult because of the avoidance of phrenic nerve and sinus node injury. Omnipolar mapping technology (OT) could enhance the efficacy of SVC isolation, requiring fewer RF applications and reducing procedure duration.

Abbreviations: CM, conventional method; OT, omnipolar mapping technology; RF, radiofrequency; SVC, superior vena cava; SVC, superior vena cava.

In the OT group, the duration required for mapping was notably extended in comparison to the CM group (6.6 ± 2.8 min vs. 5.3 ± 3.2 min, *p* = .049). Furthermore, the duration of the radiofrequency procedure in the OT group was notably less than that in the CM group (9.6 ± 6.8 min vs. 14.3 ± 6.8 min, *p* = .007). There was no significant difference in the total procedure time for SVC isolation between the two groups. The OT group necessitated a reduced number of RF applications for SVC isolation compared to the CM group (13.6 ± 6.0 vs. 19.8 ± 10.9, *p* = .046). Additionally, the OT group used less RF energy (7104 ± 4410 J vs. 10,088 ± 5760 J, *p* = .039). No significant variation was observed in the fluoroscopic times needed for SVC isolation between both groups. However, the overall absorbed dose was notably lower in the OT group (69.6 ± 47.6 mGy vs. 90.3 ± 30.3 mGy, *p* = .023). A single patient from each group experienced a PN injury, with no additional complications noted.

### 3.3 RA‐SVC conduction block line

In the OT group, the average count of mapping points was recorded as 931 ± 335. Eighteen patients exhibited the RA‐SVC conduction block line, while seven patients did not. RA‐SVC conduction block line was classified into the following three types based on the report of Tanaka and his colleague.^7^ (1) Type I: An oblique straight line of conduction block running from the lower lateral wall to the higher anterior wall. (2) Type J: The conduction block line traversed from the lower posterolateral wall, reaching its apex at the anterior wall, and concluded at the elevated mid‐septal wall. (3) Type U: This refers to a block line that extends from the lower posterior region to the lower posteroseptal area, traversing the higher anterior wall around the junction of the RA‐SVC. The distribution of block line types was as follows: I type accounted for 40%, J type for 20%, U type for 12%, and no block line for 28%. In every instance, the comparison between bipolar and omnipolar mapping revealed no significant disparity in terms of both the SVC sleeve length (32.9 ± 10.6 mm vs. 30.5 ± 10.9 mm, *p* = .16) and the block line (15.6 ± 15.3 mm vs. 16.4 ± 17.0 mm, *p* = .91).

### 3.4 Differences of sinus node locations between omnipolar and bipolar mapping

**FIGURE 3:**
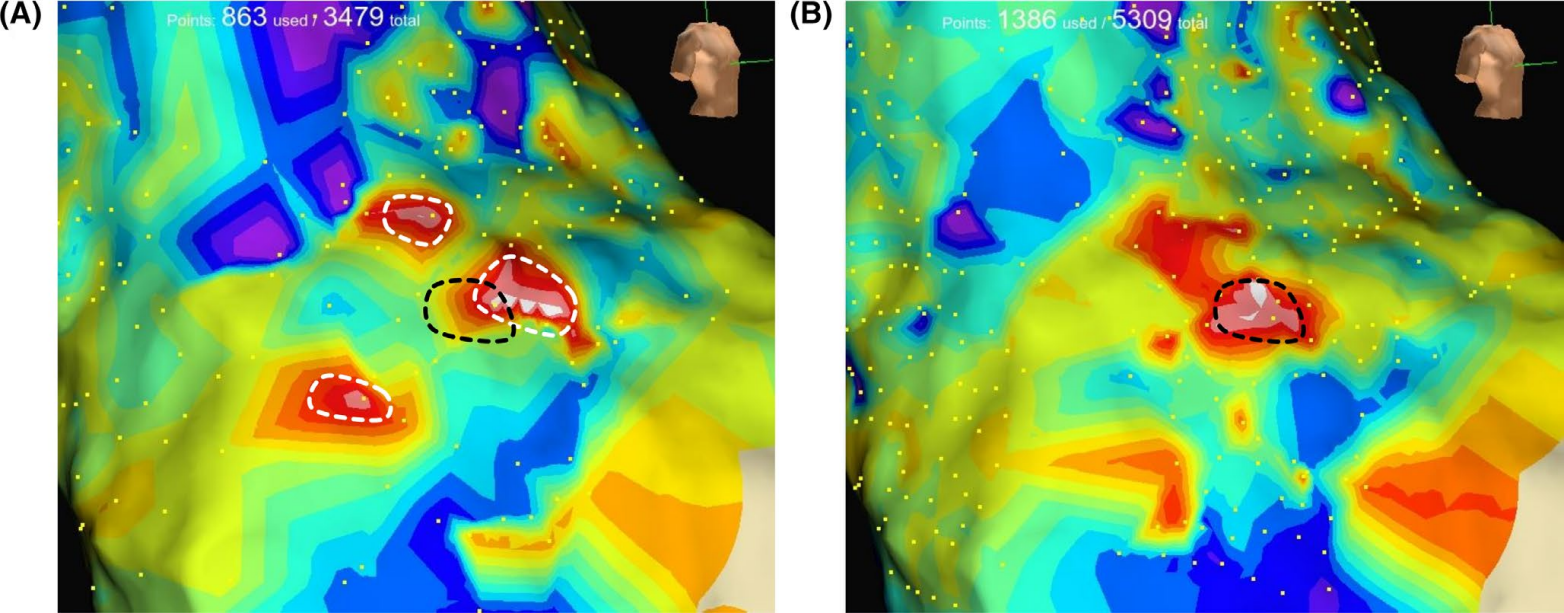
Representative case of electroanatomical maps of the RA‐SVC in the OT group. Intracardiac electrograms were concurrently gathered using either bipolar (A) or omnipolar (B) mapping techniques. The region encircled by white and black dotted lines illustrates the earliest activation site in bipolar and omnipolar maps, respectively. OT, omnipolar mapping technology; RA, right atrium; SVC, superior vena cava.

We also investigated the differences between omnipolar and bipolar mapping in 25 patients in the OT group. In 3 of 25 patients, OT clearly identified the location of SN that was not identified by bipolar mapping. Figure 3 shows a representative case in which the dereference in the location of SN between the OT and bipolar mapping. The earliest activation site was identifiable using the OT yet remained undetectable when employing bipolar mapping. Moreover, the initial activation sites identified in the bipolar mapping were dispersed across the three locations surrounding the primary activation sites in total polarity mapping (Figure 3A,B).

## 4 DISCUSSION

The present study yields three significant findings. (1) The efficacy of SVC isolation, identified by OT, was comparable to previously reported other high‐resolution mapping techniques. The selective ablation of the SVC‐RA connection using the OT resulted in a comparable success rate of SVC isolation to that of the conventional method based on the anatomical approach. (2) The OT facilitated SVC isolation more efficiently, requiring fewer RF applications and reducing procedure time compared to the conventional method. (3) The OT demonstrated a higher degree of precision in identifying the location of the SN compared to bipolar mapping, while the type of RA‐SVC conduction block line and the SVC sleeve length were similar for both OT and bipolar mapping.

This study represents the first instance of visualizing the RA‐SVC conduction block line using the OT. Tanaka and his associate previously introduced a method for illustrating RA‐SVC conduction block using the Rhythmia HDx electroanatomic mapping system (Boston Scientific). They defined the conduction block as a line where the spectrum encompassed ≥ three colors within a short span.^7^ Conversely, Inagaki et al. employed the early‐meets‐late feature of the CARTO3 mapping system (Biosense Webster, Inc., CA, USA) to depict the RA‐SVC conduction block as a white line on the local activation map.^11^ Both studies used local bipolar intracardiac electrograms gathered with a multipolar mapping catheter to describe RA‐SVC block lines. However, it remained uncertain whether these methods would yield visualizations comparable to the OT. We conducted a comparison of the visualized block lines. The OT and bipolar mapping were conducted concurrently, confirming that the positions and lengths of the block lines were consistent across both mapping techniques.

In this study, we observed that seven patients (28%) had no block line, whereas Tanaka et al. reported a higher percentage of 50.5%.^7^ According to their study, patients with nonblock lines were younger than those with block lines. In contrast, the mean age of the patients in our study was 68.0 years, which is older than the population in their research. This age variation may have contributed to the disparity in the proportion of block lines observed between the two studies. Although the lower proportion of nonblock lines in our study may have slightly influenced the results, the number of RF applications, total RF energy, and total RF time in the OT group did not differ compared to previous studies. Therefore, the impact of this difference in the proportion of nonblock lines is considered negligible.

Goya et al. have presented their findings on the anatomically guided circumferential isolation of the SVC using a circular mapping catheter. They showed that the number of breakthroughs from the RA to the SVC was 1.4 ± 0.5 per patient, and the mean number of RF applications for SVC isolation was 4.0 ± 1.0 per patient.^12^ In the present study, we needed more RF applications for SVC isolation (13.6 ± 6.0 and 19.8 ± 10.9 in the OT and CM groups, respectively). The observed results could potentially be ascribed to the ablation technique employed in the current research. In the CM group, we employed a circumferential ablation technique, while in the OT group, we ablated only nonblock line areas as opposed to a point‐by‐point ablation at the earliest activation site, as identified by a circular mapping catheter. Given that selective point‐by‐point ablation for LA and PV connection has been associated with late reconnection of LA and PV, we opted for circumferential SVC isolation.^13^ This approach may have contributed to an increase in RF applications in the OT and CM groups. Previous research using high‐density mapping for SVC isolation has indicated an average of 10 to 15 RF applications required for isolation. Our findings align closely with these reported averages.^7^, ^8^, ^9^, ^11^

PN injury during SVC isolation is a significant complication, with prior research indicating its occurrence in roughly 5% of patients undergoing SVC isolation.^8^, ^14^ We hypothesized that the outcomes related to safety would be superior in the OT group compared to the conventional group. This is because of the OT group's ability to achieve isolation with fewer RF applications and reduced energy usage. Despite our efforts to reduce the RF energy and duration at the PN capture site, we observed PN injury during the SVC isolation in one patient, representing 4.0% of each group. All patients who experienced PN injury necessitated ablation at the sites of PN capture. In the OT group, over 50% of the cases exhibited no block line at the PN capture site, potentially leading to a PN injury in one patient. In instances involving U‐type and J‐type block lines, we consciously avoided administering radiofrequency applications to the phrenic nerve capture site, thereby mitigating the risk of phrenic nerve damage.

Determining the location of the SN during SVC isolation is crucial to preventing damage to the SN. In the present study, OT identified the location of SN that was not detected by bipolar mapping. Several studies have demonstrated the superiority of OT over bipolar mapping in electroanatomical mapping. Karatela et al. showed that OT yielded a superior voltage and point density compared to bipolar mapping using a high‐density wave algorithm.^15^ Additionally, another study revealed that omnipolar signals are virtually unaffected by catheter orientation, allowing LAT mapping, which is susceptible to minor timing discrepancies, to be performed with greater consistency.^16^ We assume that the failure of bipolar mapping to identify the earliest excitation sites in our study is due to bipolar blindness, which prevents the detection of activation wavefronts moving perpendicular to an electrode pair.

In this study, 62 of 330 (18.8%) patients with AF underwent SVC isolation. This proportion was higher than the reported prevalence of SVC foci, which is approximately 5%.^4^ This discrepancy is attributable to the criteria for SVC isolation employed in this study. Higuchi et al. reported that an SVC sleeve length of 30 mm or more and SVC potentials exceeding 1.0 mV indicate that the SVC can serve as a source of AF.^17^ In this study, a preventive SVC isolation strategy was applied in cases undergoing a second or subsequent ablation procedure if the SVC sleeve length was sufficient, which contributed to the higher proportion than the previous study. In this study, the proportion of patients who actually developed AF from SVC was approximately 3%, which was similar to previous studies.

The study concluded prematurely, failing to achieve the intended sample size, attributable to a recruitment rate that fell short of expectations. Despite potential statistical power reduction caused by the sample size, the current study demonstrated that SVC isolation using OT is not inferior to the conventional method. We exclusively evaluated the immediate success rate of SVC isolation. Therefore, it is yet to be conclusively established whether the OT or CM method is more effective for long‐term maintenance of RA‐SVC isolation. There is a need for long‐term follow‐up to assess efficacy and safety outcomes. Owing to the open‐label design of this study, the potential for investigator bias influencing modifications to procedural aspects cannot be ruled out.

## 5 CONCLUSION

This is the first study to randomly compare conventional ablation of the RA‐SVC junction based on angiography to selective ablation of the SVC‐RA connection using OT. Selective ablation of the SVC‐RA connection using the OT resulted in effective SVC isolation in shorter procedural time and with fewer RF applications compared to conventional treatment methods.

## CONFLICT OF INTEREST STATEMENT

The authors declare no conflicts of interest.

## ETHICS STATEMENT

Approval of the Research Protocol: The study received approval from the Institutional Ethics Committee of Hiroshima University's Graduate School of Biomedical Science (approval no. C2021‐0339, registered on September 30, 2020). It was conducted in strict adherence to the principles outlined in the Declaration of Helsinki. Informed Consent: All participants furnished their written informed consent. Animal Studies: N/A.
